# Sensor-based algorithmic dosing suggestions for oral administration of levodopa/carbidopa microtablets for Parkinson’s disease: a first experience

**DOI:** 10.1007/s00415-019-09183-6

**Published:** 2019-01-18

**Authors:** Ilias Thomas, Moudud Alam, Filip Bergquist, Dongni Johansson, Mevludin Memedi, Dag Nyholm, Jerker Westin

**Affiliations:** 1 Department of Micro-data Analysis, Dalarna University, 79 131 Falun, Sweden; 2 Department of Clinical Neuroscience, Institute of Neuroscience and Physiology, Sahlgrenska Academy, University of Gothenburg, 413 90 Gothenburg, Sweden; 3 School of Business, Orebro University, 702 81 Örebro, Sweden; 4 Department of Neuroscience, Neurology, Uppsala University, 751 85 Uppsala, Sweden

**Keywords:** Levodopa, Parkinson’s disease, Algorithmic suggestions, Sensor data, Oral medication

## Abstract

**Objective:**

Dosing schedules for oral levodopa in advanced stages of Parkinson’s disease (PD) require careful tailoring to fit the needs of each patient. This study proposes a dosing algorithm for oral administration of levodopa and evaluates its integration into a sensor-based dosing system (SBDS).

**Materials and methods:**

In collaboration with two movement disorder experts a knowledge-driven, simulation based algorithm was designed and integrated into a SBDS. The SBDS uses data from wearable sensors to fit individual patient models, which are then used as input to the dosing algorithm. To access the feasibility of using the SBDS in clinical practice its performance was evaluated during a clinical experiment where dosing optimization of oral levodopa was explored. The supervising neurologist made dosing adjustments based on data from the Parkinson’s KinetiGraph™ (PKG) that the patients wore for a week in a free living setting. The dosing suggestions of the SBDS were compared with the PKG-guided adjustments.

**Results:**

The SBDS maintenance and morning dosing suggestions had a Pearson’s correlation of 0.80 and 0.95 (with mean relative errors of 21% and 12.5%), to the PKG-guided dosing adjustments. Paired *t* test indicated no statistical differences between the algorithmic suggestions and the clinician’s adjustments.

**Conclusion:**

This study shows that it is possible to use algorithmic sensor-based dosing adjustments to optimize treatment with oral medication for PD patients.

## Introduction

Parkinson’s disease (PD) is a movement disorder that is characterized by the cardinal symptoms: bradykinesia, tremor, rigidity and postural instability [1]. There is currently no cure for PD and the reasons for the disease’s onset are not known. What is available to PD patients are treatment options (with the most effective treatment being levodopa intake) that limit the disease’s symptom manifestations and to some extent restore motor functions.

The process of titrating the dosing schedule for PD patients can be vaguely categorized into three stages, along with disease progression. In the first stage, physicians prescribe oral administration of levodopa using a generic dosing schedule but as the disease progresses the patients start experiencing shortening of medication effect, wearing-off fluctuations, and sometimes dyskinesia (manifestation of involuntary movements—attributed to overmedication) [2, 3]. In those cases, individually tailored dosing routines are given, as a more advanced dosing strategy (second stage). Those dosing schedules may include a morning dose (the first dose of the day) and subsequent maintenance doses (of a set amount), throughout the day, at specific time points. The need for morning dose is patient-specific, because of the sleep benefit phenomenon [4], where there is less immediate need for levodopa in the morning for some patients.

Individualizing the dosing routines, however, presents difficulties. Patient diaries [5] and limited information during clinical visits will often not provide the physician with the information necessary to optimize dosing routines appropriately. It is not unusual for the patients on individualized treatment to experience either hour-long periods of “off”, i.e. no effect from medication, or “dyskinesia”, because of ill-adjusted dosing schedules. This can become problematic for the patients, who then might require advanced non-oral therapies [6, 7] (third stage).

Before that becomes necessary, the best option for patients that can be managed with oral medication (or for patients that cannot receive advanced non-oral therapy), would be to further adjust the dosing routines to minimize the periods of sub-optimal treatment. This requires well-informed dosing adjustments. To that end, sensor-based systems that allow for objective measurements of the patients’ motor status have been developed and become increasingly popular. Over the past years studies about symptom monitoring through wearable sensors have been published [8, 9] and commercial products continue to become available, such as the Kinesia™ systems [10] and the Parkinson’s KinetiGraph™ (PKG) [11] that produces scores for bradykinesia, tremor, dyskinesia and motor fluctuations. PKG is an accelerometry based system which generates continuous movement patterns, indicating both dyskinesia and bradykinesia, and allows the clinicians to assess motor fluctuations and evaluate treatment efficacy. Furthermore, new formulations that allow for precise dosing have been developed, such as the microtablets of levodopa/carbidopa (Flexilev^®^), which are specifically designed for optimization of levodopa dosage [12], in steps of 5 mg levodopa. Even though steps have been taken in the direction of precise medicine, there is a lack of dose optimization algorithms designed for oral administration of levodopa. The main aim of this paper is to propose and describe such a dosing algorithm used for a novel dosing system design. This system uses objective measurements as input to a simulation algorithm that provides individually tailored dosing schedules. To the best of the authors’ knowledge, this is the first attempt at developing this type of medical decision support for oral treatment in Parkinson’s disease.

## Materials and methods

### Patient-specific dosing algorithm

In Thomas et al. [13] a dosing algorithm that determines individual dosing suggestions for continuous infusion of levodopa was described. In that algorithm the dosing suggestions were limited to an optimized infusion rate for a carbidopa–levodopa infusion device. As the treatment strategies for oral and continuous levodopa dosing have different requirements, mainly the need for dosing schedules, an advanced algorithm was designed specifically for oral administration of levodopa. This dosing algorithm is knowledge-driven and was developed in collaboration with two movement disorder expert neurologists, FB and DN, aiming to imitate informed clinical practice dosing decisions. It derives dosing schedules for a 16-h day and suggests a morning dose and maintenance doses at specific time points. The maintenance doses are the same within each dosing schedule but the morning dose might be different, usually higher than the maintenance doses, to more rapidly reach pharmacologic steady-state, as in clinical practice. For each patient the algorithm examines different dosing frequencies (the time between two successive doses) via a simulation study.

The aim of the process is to find the combination of morning and maintenance dose that minimizes the period of “off” and “dyskinesia” (maximizing the time of optimal motor functions) for each dosing frequency. The simulations are run on the treatment response scale (TRS), which ranges from − 3 to + 3, with negative values indicating to “off”, positive values to dyskinesia, and values close to 0 to the optimal status [14]. This scale provides a holistic way to monitor the disease as all three states are represented and it is possible to observe the transition into different states in a continuous fashion, which facilitates the simulations. The input to the algorithm are individual patient models (a detailed description of how individual models are built is given in “Sensor-based dosing system”). For every patient the specific model derived dose–effect curves are produced for different combinations of morning and maintenance dose and at different dosing frequencies.

**Fig. 1:**
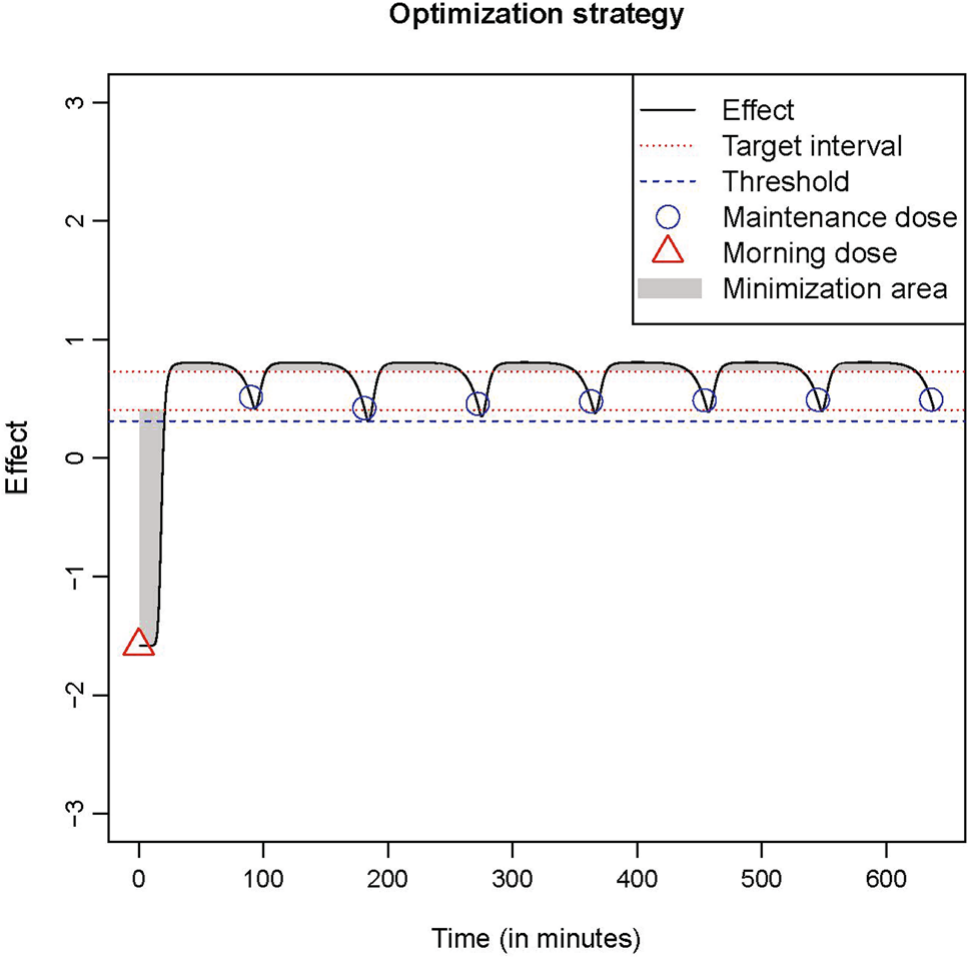
Dose optimization strategy of the algorithm for an example patient. The target range is indicated by the red lines

Before initiating the above simulation process the dosing frequencies to be examined are selected together with the target state (a value between − 3 and + 3) for each patient. Setting a positive value close to 0 as target is usually preferred to avoid fluctuation between two does. To avoid overmedication and keep the dose at acceptable levels, a target range is set around the target value which ensures that there is some reduction in effect before the next dose is taken, thus balancing the dose to a value that is sufficiently large but also low enough. For every dosing frequency a combination of maintenance doses and morning doses are simulated, the dose–effect curve values are stored for each combination, and the best one is selected. The best combination of morning and maintenance doses is defined as the one that minimizes the time the simulated dose–effect curve is outside the target-range (Fig. 1). Furthermore, there is a fluctuation criterion, which determines when a dose is acceptable or not. When the simulated effect reaches a maximum in a time point between two doses, it is not allowed to drop below a specific threshold before the next dose is taken. That ensures that the dose is large enough and the patients do not experience substantial fluctuations in a normal day, which is the goal with dose optimization of oral administration of levodopa in standard clinical practice. The best combination is, therefore, the one that produces the minimum area outside the target-range and also meets the fluctuation criterion.

A graphical illustration of a dose optimization example can be seen in Fig. 1, where, the simulated dose–effect of an individual model is displayed in response to morning and maintenance doses. In this example the target was set at 0.5, the target-range was between 0.7 and 0.4, and the threshold was at 0.3, meaning the patient’s status was not allowed to drop below that value. In this simulation the dose frequency is 90 min, and as shown in the figure a dose is simulated every 90 min. The algorithm was evaluated during a clinical experiment as part of sensor-based dosing system (SBDS), and more details about its implementation are given in “Application of the SBDS”. A detailed description of the SBDS and the individual model fitting process is given in the next section.

### Sensor-based dosing system

As mentioned earlier, the input to the dosing algorithm are individual patient models. In Thomas at al [13]. a method to fit individual dose–effect models for levodopa infusion in PD was developed based on a pharmacokinetic–pharmacodynamics (PKPD) model [15]. The PKPD model is a system of mathematical equations that describe the process from dose intake to absorption and distribution until manifestation of an effect, in response to the dose, on the TRS. The PKPD model is described in the following equations:

1 $$\frac{{{\text{d}}{a_0}}}{{{\text{d}}t}}={\text{Inf}} - {k_a} \times {a_0},$$

2 $$\frac{{{\text{d}}{a_1}}}{{{\text{d}}t}}={\text{BIO}} \times {k_a} \times {a_0} - \left( {\frac{{{\text{Q}}+{\text{CL}}}}{{{V_1}}}} \right) \times {a_1}+\left( {\frac{Q}{{{V_2}}}} \right) \times {a_2}+{\text{Rsyn,}}$$

3 $$\frac{{{\text{d}}{a_2}}}{{{\text{d}}t}}=\left( {\frac{{\text{Q}}}{{{V_1}}}} \right) \times {a_1} - \left( {\frac{Q}{{{V_2}}}} \right) \times {a_2},$$

4 $$\frac{{{\text{d}}{c_{\text{e}}}}}{{{\text{d}}t}}={\text{kEO}} \times \left( {\frac{{{a_1}}}{{{V_1}}} - {c_{\text{e}}}} \right),$$

5 $$E={\text{BASE}}+\frac{{{E_{{\text{max}}}} \times c_{{\text{e}}}^{\gamma }}}{{c_{{\text{e}}}^{\gamma }+{\text{EC}}{{50}^\gamma }}}.$$

The parameter description of the PKPD model is given in Table 1.

**Table 1:** Parameter description of Eqs. (1)–(5)

Inf	Intestinal levodopa infusion rate (mg/min)	+
*a*_0_	Amount in first compartment (mg)	+
*a*_1_	Amount in second compartment (mg)	+
*a*_2_	Amount in third compartment (mg)	+
${k_a}$	Absorption rate (1/min)	1/TABS^a^
TABS	1/${k_a}$, absorption time constant (min)	28.5^a^
${\text{kEO}}$	Effect rate (1/min)	1/TKEO^a^
TKEO	1/${\text{~kEO}}$, effect time constant (min)	21^a^
BIO	Bioavailability	0.88^a^
*Q*	Intercompartmental clearance (L/min)	0.58^a^
*V*_1_	Volume in first compartment (L)	11^a^
*V*_2_	Volume in second compartment (L)	27^a^
CL	Clearance rate (L/min)	0.52^a^
Rsyn	Endogenous levodopa synthesis rate (mg/min)	0.01^a^
Ce	Concentration in the effect compartment (mg/L)	+
EC50	Concentration at 50% effect (mg/L)	1.55^a^
gamma	Sigmoidicity factor	11.6^a^
BASE	Baseline effect	X
*E*_max_	Change from baseline effect	X
*E*	Effect ranging from − 3 to + 3	+

+, estimated thought the equations, given the parameter values; *X*, patient-specific values

^a^Population parameter values as seen in Westin et al. [11]

**Fig. 2:**
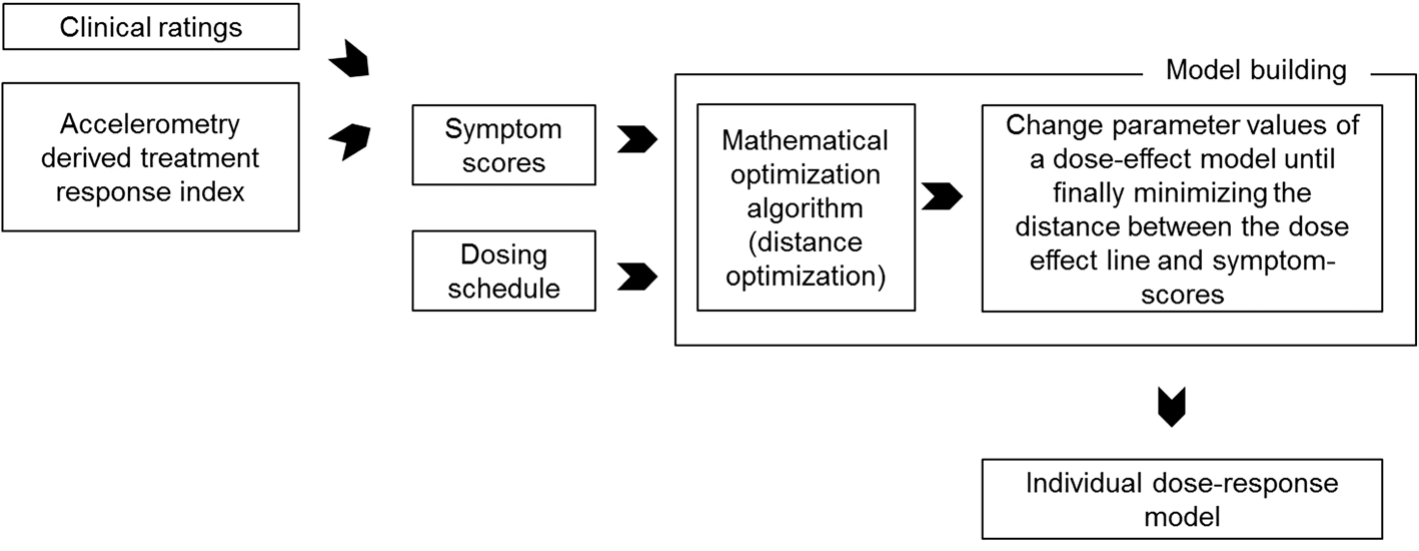
Schematic illustration of the individual model fitting process

The individual models were fitted by altering a subset of the parameter values of the population PKPD model, using the dosing information and fitting patient-specific dose–effect curve to a series of clinical observations, through least squares optimization [13]. The parameters altered are: V1, CL, TKEO, EC50, gamma. Furthermore, BASE and Emax are fixed respectively to the lowest and highest TRS values observed during an observation period for a specific patient. An illustration of the process using either clinical or objective ratings is shown in Fig. 2. What characterizes an individual patient model are the specific PKPD parameters that are estimated during the optimization process. More details of the individual model fitting process are found in [13].

As the model-fitting process requires individual ratings, the use of wearable sensors to extract the necessary information was explored. This was done to investigate whether this algorithm could be embedded into a sensor-based system. It is also possible to use this algorithm with clinical ratings as input. However, these are not usually available in real time and their acquisition requires the presence of an expert neurologist. Embedding the algorithm into a SBDS presents the opportunity of making dosing suggestions directly from patient-derived sensor data without the need for interpretation by a clinician.

In Thomas et al. [16], the use of a hand pronation supination test for automated objective scoring of Parkinson’s disease symptoms was described. The patients wore commercial 6-degrees of freedom sensors on both wrists (Shimmer3 sensors) during the test, which was performed for a 20 s period for each hand. Spatiotemporal features from the sensor readings were extracted (88 features) and principal component analysis was performed on the features. Finally, six principal component were used as predictors in a support vector machine (SVM) model that was trained for regression, predicting the patients’ motor status on the TRS [16]. The SVM model’s predictions had good clinimetric properties and high correlation (0.82 in a tenfold cross-validation setting) to clinical ratings. More details about the signal processing and the data mining methods applied can be found in [16].

**Fig. 3:**
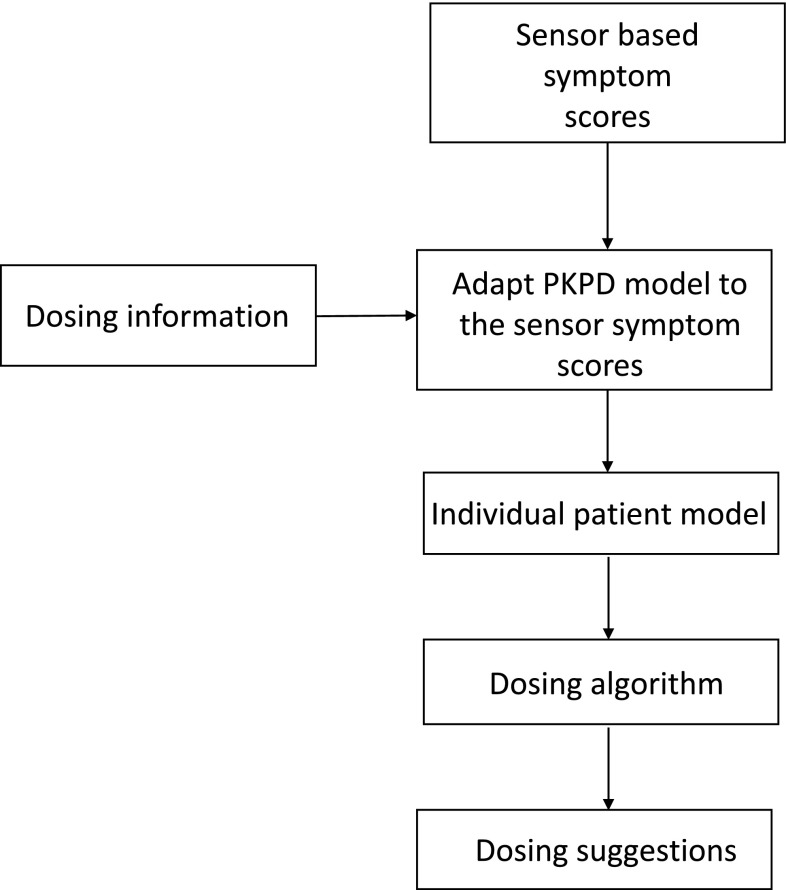
System flow-chart. The inputs to the model builder are ratings from sensor ratings and individual dosing information (Step 1). The model builder uses the dosing

The extracted objective ratings on TRS [16] were used to fit individual patient models [13], which were then used as inputs to the dosing algorithm. A graphical illustration of the SBDS can be seen in Fig. 3.

### Clinical study description

The feasibility of using the dosing algorithm as part of the SBDS was evaluated during a clinical observational study. In Johansson et al. [17], a clinical study for dose optimization of oral administration of levodopa was described. In that study, which was a follow-up to the one described in Senek et al. [18], 31 patients participated in a single-center longitudinal observational clinical study at Sahlgrenska University Hospital in Gothenburg, Sweden, between August 2016 and February 2017. The study design was approved by the regional ethics review board in Gothenburg and the participants had given written consent to the study, in accordance with the Helsinki declaration. The patients recruited were in need of frequent dose administration (dosing frequency less than 4 h). The study consisted of three patient visits to the clinic, with a 2-week period between each visit.

During the first visit the patients’ old dosing schedules were converted to the microtablet equivalent [19] and the patients were given a PKG device that they wore for a 6 day period before the second visit to the clinic. During the second visit FB evaluated the PKG readings from the previous week and adjusted the dosing schedule of the patients. The effect of the dosing adjustments were evaluated 2 weeks later at the last visit.

Of the 28 (there were 3 screening failures) patients that completed the first visit, 25 completed the first two visits and 24 completed all three visits. In Table 2 the population characteristics of the 25 patients that completed the single dose experiments can be seen. Detailed information about the study design, dosing optimization, and outcomes can be found in [17].

**Table 2:** characteristics of the PD participants in the single-dose experiments

	Sex	Median age in years (range)	Mean BMI as kg/m^2^ (range)	Median years from diagnosis (range)	Median years with motor fluctuations (range)
Patients	15 males 10 females	68 (58–82)	25.2 (20.8–35.4)	10 (4–30)	4 (1–20)

### Application of the SBDS

The SBDS was applied during the patients’ second visit to the clinic. At that time the patients put on the Shimmer3 sensors and performed pronation-supination tests at a pre-determined time schedule as in [18]. The tests were performed before and following a single dose of levodopa/carbidopa (120% of the normal morning dose), after a 12 h wash-out period during the night. A single dose allowed the patients’ status to go from a baseline value to a peak effect back to the baseline value (impulse response), making the identification of individual patient models possible [20].

The individual models (the estimated patient-specific parameters of the PKPD dose–effect model) were used as input to the dosing algorithm which had two settings for the target-range on the test day, depending on the maximum TRS the patients demonstrated. If it was higher than 0, the target-range would be set as a percentage value of their maximum TRS. For the patients where the maximum TRS was lower than 0.05, or negative, the target-range was set in absolute terms, since the percentage approach calculation was not suitable in those two cases. In the first case a percentage value of a score close to 0 would also be 0, thus not allowing for any wear off in effect (leading to overmedication). In the second case, a percentage value of negative scores would only allow for the fluctuations to get larger for the lower effect values. This adjustment to the design was necessary since the sensor-based TRS ratings would not produce positive values for some patients. The fluctuation criterion value was set as 0.5 points of the TRS.

The dosing frequencies examined by the SBDS were per 90 min (although in some patients the dosing frequencies would be even shorter) to per 240 min (as 4 h was the maximum dosing frequency according to inclusion criteria) and there was a 10 min increment between every simulation. The algorithm performed the simulations for each dosing frequency, starting from a minimum dose of 0 mg of levodopa (no dose) to a maximum dose that was patient-specific, depending on the dose they received during the test day (the maximum was 300 mg of levodopa for maintenance doses and 400 mg for morning dose). This procedure was performed for all dosing frequencies and the algorithm would only suggest one dosing combination (morning and maintenance dose) for each, the one with the minimum area outside the target-range that satisfied the fluctuation criterion. For a 90–240 min dosing frequency selection with 10 min increments there would be 16 dosing frequencies investigated and for each of them there would be a single combination of morning and maintenance dose derived. In this example, 16 different dosing schedules for each patient would be derived and about 120,000 (301 × 401) dosing combinations would be simulated for every dosing schedule.

The suggestions of the SBDS were compared to the PKG-aided dosing adjustments of the second visit, for the same dosing frequency. For example, if a schedule that required dosing every 100 min was selected by FB based on the PKG recordings, those dosing adjustments would be compared to the dosing adjustments suggestions of the SBDS for the 100 min frequency (which is the equivalent of running the simulation for only one dosing frequency and producing one dosing schedule).

## Results

In total, sensor readings for all 25 patients were obtained, but appropriate individual models could only be fitted for 19 patients. One patient could not perform the hand rotation task, one patient had limited response to levodopa during the test day, and for four patients the algorithm predictions misrepresented the dose–effect behavior at the day of the trial. For those four patients, the series of sensor index scores did not represent a “normal” dose–response curve, i.e the values did not go from baseline to a peak effect and then back to baseline. Results from the remaining 19 patients are presented here, for nine of which one test occasion was removed as outlier in the model fitting process and for one patient two test occasions were removed as outliers. Outliers were identified as scores that had either a sudden drop in value after the onset of effect, or displayed a sudden spike in effect after wearing off started to demonstrate. Sudden motor fluctuations do happen, but cannot be accommodated by the current PKPD models. That is why these values were selected as outliers, even though they might not be in clinical practice. The outliers were removed based on a visual inspection of the TRS scores.

**Fig. 4:**
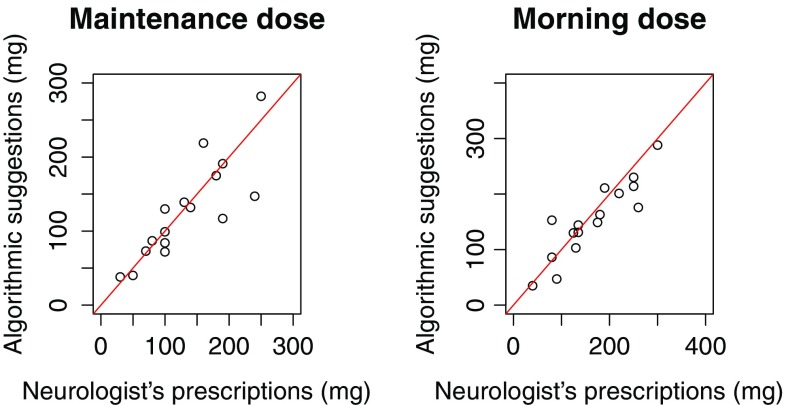
Visual comparison of the algorithms suggestions compared to the physician’s choices for the same dosing frequency

The Pearson’s correlation of the maintenance dosing suggestions with the PKG-guided prescriptions, for the same frequency, was 0.80, and the mean relative error of the predictions was 21%. For the morning dose the Pearson’s correlation was higher (0.95) with a lower mean relative error, 12.5%. The results for the maintenance dose are influenced by three values that have high relative error. In Fig. 4 a visual comparison of the suggestions, for the frequency of the PKG-guided choice, is presented.

To test the similarity of the algorithmic suggestions to the PKG-guided choices, a paired sample *t* test was performed to check if there were significant statistical differences. The test was conducted with the null hypothesis that the dosing values were not significantly different (mean difference is 0). The results of the *t* test had a *p* value of 0.61 for the morning dose and 0.79 for the maintenance dose, meaning that the null hypothesis could not be rejected in any case. Based on these results, there was no evidence in the data that the algorithmic dosing suggestions were different from PKG-guided adjustments. These dosing adjustments improved the patients’ motor functions [17]. Since the suggestions of the algorithm were mostly similar, there is reason to suggest that had the patients received the SBDS suggestions, they would have demonstrated similar improvement.

## Discussion and conclusions

The necessity of a SBDS emerges from a continuous discussion raised also by Espay et al. [9] and Titova et al. [21]. Espay et al. argue that wearable sensors should be used for precision medicine, and that algorithms could be developed to generate specific recommendations. The recommendations of the proposed SBDS in this study are symptom specific, since individually derived sensor index values are used [16].

The algorithm can be described as one that follows a dose-fractionation principle, allowing about 5–15 doses per day [22] and is designed specifically for patients that experience levodopa-induced dyskinesia (LID). The SBDS does not consider combinations of different medications but is restricted to oral levodopa/carbidopa. Since about a third of the patients will experience LID after about 7 years with the disease, the proposed SBDS is relevant to the PD community. To the authors’ best knowledge, similar patient-specific dose suggestion algorithms are not available, and the introduction of one in this study could inspire a more focused effort to develop such applications.

This algorithm mimics well-informed clinical decision-making. Such a design was chosen as a first attempt to investigate the feasibility of algorithmic dosing suggestions, and different designs, such as pattern recognition algorithms or mathematical optimization algorithms, could and actually should be explored in the future. The feasibility of using the method was tested during a clinical study where it was found that the suggestions of the method had high correlation to the supervising physician’s prescriptions, which were not subjective but PKG-guided. At the next stages, the algorithm could be integrated into an interactive platform such as the one in [23], so that the suggested method could be used in a home environment setting by the patients between or before clinical visits.

However, there were limitations, mainly the need to manually remove outliers before the model fitting process. At least one sensor rating was removed for ten patients, out of 19 reported in this study. It should also be pointed out that FB had input on the design of the algorithm (setting targets and ranges) as well as the patients’ dosing adjustments and the generalizability of the results might, therefore, be somewhat limited. This is why it is important to consider that the PKG readings were the basis of the adjustment decisions and FB was merely the facilitator between patient and PKG-recording. Since the algorithm was designed to mimic dosing optimization as it is performed in clinical practice, the similarity of the algorithm output and the clinician’s dose suggestions is a positive outcome.

It can be concluded that given a high enough dose to produce a dose–effect and the ability of the patients to perform motor tasks the SBDS is quite robust when producing dosing suggestions. There were certainly occasions where individual models would fail to provide useful information. In these cases, however, the limitation of the method is also attributed to the inability to make accurate predictions with the SVM model [16], therefore, this study evaluates not just the performance of a dosing algorithm, but of the SBDS. The results are quite promising when appraising the reality that the neurologist had continuous sensor recordings for a 6 day period from the PKG, whereas the algorithm evaluated only 8–12 test occasions, during a 4 h period, to make the dosing suggestions. In should be noted, however, that for patients where the dose effect profile is misrepresented the dosing suggestions could be unsuitable, worsening the quality of life of patients. This is why, at the current stage, an intermediate step to confirm the model fit is deemed necessary.

The future work will focus on addressing the limitations of the current version of the SBDS and automate the model fitting process including algorithmic exclusion of outliers. The current comparison with clinical decisions was made to provide proof of concept, but future development should have the broader aim of optimizing patient outcome instead.
